# Composition Descriptors and Cultivar Transferability in Machine-Learning Models of Ultrasonication-Induced Functional Properties of Rice Flour

**DOI:** 10.3390/foods15132268

**Published:** 2026-06-24

**Authors:** Hyeonbin Oh, Jung-Hyun Nam, Bo-Ram Park, Kyung Mi Kim, Ha Yun Kim, Yong Sik Cho

**Affiliations:** 1 Department of Food Sciences, National Institute of Crop and Food Science, Rural Development Administration, Wanju-gun 55365, Jeollabuk-do, Republic of Korea; irnark@korea.kr (H.O.); wwre44@korea.kr (J.-H.N.); bboram27@korea.kr (B.-R.P.); kimkm@korea.kr (K.M.K.); khy0617@korea.kr (H.Y.K.); 2 Department of Agricultural Engineering, National Institute of Agricultural Sciences, Rural Development Administration, Wanju-gun 55365, Jeollabuk-do, Republic of Korea

**Keywords:** gelatinized rice slurry, ultrasonication, machine learning, cultivar variation, SHAP, transferability

## Abstract

Flow-cell ultrasonication of gelatinized rice flour slurries alters cultivar-dependent water solubility, viscosity, and retrogradation of pregelatinized rice flour, properties important for plant-based beverages and convenience foods. We tested whether cultivar-level composition descriptors, amylose, protein, and fiber, can represent cultivar-associated variation in ultrasonication responses while separating process-only prediction, within-domain cultivar representation, and unseen-cultivar transfer. Six rice cultivars were processed across nine amplitude-time combinations and two slurry concentrations. Water solubility index, apparent viscosity at a shear rate of 50 s^−1^, and setback viscosity were modeled using ElasticNet, partial least squares regression, support vector regression, random forest, and extreme gradient boosting. Three input formulations were compared: process variables alone, process variables plus composition descriptors, and process variables plus cultivar identity. Repeated nested group cross-validation showed insufficient process-only prediction and substantial improvement from composition descriptors. Within-domain validation showed comparable composition-descriptor and cultivar-identity performance under nonlinear algorithms. However, because cultivar identity is undefined for absent cultivars, leave-one-cultivar-out transfer of the composition-descriptor model remained uncertain. Cross-fitted Shapley additive explanations showed predictions used process and composition variables. For the validated cultivar-process domain, this approach can screen cultivar-process combinations for beverage and convenience-food applications, but replacing categorical source identifiers with continuous descriptors requires explicit transfer validation.

## 1. Introduction

Among plant-based milk alternatives, rice-based beverages are valued for their mild flavor, low allergenicity, and suitability for consumers seeking dairy-free options [1,2]. However, the formulation of these beverages remains limited by the intrinsic properties of rice flour. Native or conventionally milled rice flour disperses poorly in cold water. It undergoes substantial changes in viscosity during heating and cooling, which can lead to sedimentation, excessive thickening, or undesirable mouthfeel in beverage-type products [3]. Viscosity can be reduced via enzymatic hydrolysis, hydrocolloid addition, and starch modification, thereby reducing sedimentation and adjusting starch functionality in rice beverages and flour formulations [4,5,6]. However, these strategies may compromise the clean-label requirement or introduce unwanted sweetness [7].

Ultrasonication has recently garnered increasing attention as a physical alternative for rice flour modification. Acoustic cavitation during ultrasonication disrupts starch granules and breaks their chains with conformational effects similar to those reported for cereal proteins [8,9,10]. In pregelatinized rice flour, ultrasonication has been reported to increase cold-water solubility and reduce paste viscosity [11]. However, cultivar-dependent functional outcomes have been reported in rice systems subjected to the same ultrasonication conditions [9,12]. These cultivar-dependent differences are associated with compositional variations, including amylose, protein, and fiber, which influence rice pasting, texture, and retrogradation behavior [13,14].

Machine-learning models involving multiple input variables have been used for rice flour classification and food quality prediction [15,16]. Nonlinear responses are plausible in ultrasonicated rice flour because the ultrasound effects depend on the treatment conditions and starch–protein structural changes [17,18]. As responses may involve linear and nonlinear interactions, multiple algorithms should be compared rather than relying on a single model class in advance [19,20]. Furthermore, the Shapley Additive exPlanations (SHAP) can be used to examine the roles of treatment conditions and cultivar composition in model predictions [21].

In this study, the ultrasonication responses of pregelatinized rice flour from six Korean Japonica cultivars were modeled using amylose, protein, and fiber as composition descriptors. These descriptors were assessed either as substitutes for cultivar labels within the six-cultivar set or as descriptors for cultivars that were not used in training. Within-domain performance was assessed by comparing process-only inputs, composition descriptors, and cultivar labels, whereas unseen cultivar transfer was assessed using leave-one-cultivar-out validation.

## 2. Materials and Methods

### 2.1. Rice Cultivars and Compositional Analysis

Six Korean Japonica rice cultivars (*Oryza sativa* L.), namely Samgwang, Saechungmu, Seolgang, Weolbaek, Chamdream, and Akibare, were procured from the National Institute of Crop Science (Wanju, Republic of Korea). Amylose content was determined using an amylose/amylopectin assay kit (K-AMYL; Neogen, Lansing, MI, USA). Crude protein content was determined via the Kjeldahl method using a nitrogen-to-protein conversion factor of 5.95, and total dietary fiber content was analyzed according to AOAC Method 985.29. All compositional analyses were performed in triplicate before processing, and the resulting cultivar-specific profiles (Table S1) were used as composition descriptors within the modeling framework described in Section 2.5. Rice grains were dry-milled at a commercial milling facility (Saerom Food, Incheon, Republic of Korea) using an air mill, and the flour fraction was passed through a 150-μm sieve and collected for subsequent experiments. Pairwise correlations among the cultivar-level descriptors were examined before modeling: amylose and fiber were moderately negatively correlated (r = −0.63), whereas the amylose–protein and protein–fiber correlations were weak (r = −0.18 and −0.11).

### 2.2. Pregelatinization and Ultrasonication

Rice flour was dispersed in distilled water at concentrations of 7% or 10% (*w*/*w*) and heated at 95 °C for 30 min in a water bath with continuous overhead stirring using a triple-bladed impeller to prepare pregelatinized slurries. Ultrasonication was performed using the recirculating flow-cell system as described previously [22]. The system consisted of a flow cell with a 100 mL working volume, mounted on an ultrasonic processor (VCX-500; Sonics & Materials, Newtown, CT, USA) equipped with a 13 mm probe, operating at 20 kHz. To minimize heat accumulation during treatment, the flow cell was jacketed and connected to a circulating chiller, (RW3-1025; Jeio-tech, Daejeon, Republic of Korea) maintaining a temperature of 4 °C. Slurries were continuously circulated through the flow cell at 500 mL/min using a peristaltic pump (TPT6035; LK Labkorea, Namyangju-si, Republic of Korea) fitted with #16 silicone tubing, and pump speed was adjusted in rpm to ensure a constant flow rate across both slurry concentrations. Sonication was initiated when the core sample temperature reached 70 °C. Treatments were performed using a full factorial design comprising two slurry concentrations (7% and 10%, *w*/*w*), three amplitude settings (40%, 60%, and 80%; reported as instrument settings), and three treatment durations (15, 30, and 45 min), resulting in 108 unique cultivar–process combinations (6 cultivars × 2 concentrations × 9 ultrasonication conditions), each prepared in triplicate (n = 324). Following ultrasonication, samples were lyophilized for 72 h, ground, and passed through a 150 μm sieve. The resulting pregelatinized rice flour samples were stored at 4 °C until further analysis.

### 2.3. Response Variable Measurements

The following response variables were evaluated: water solubility index at 25 °C (WSI), apparent viscosity at 50 s^−1^ (η_50_), and setback viscosity derived from pasting profiles (Setback). These three responses were selected a priori to represent cold-water solubility, steady-shear flow behavior relevant to beverage applications, and post-cooling reassociation, respectively. Setback was used as the representative pasting parameter because it was strongly correlated with peak, breakdown, and final viscosity (r = 0.82–0.99). WSI was determined following a modified version of the method described previously [23]. Briefly, 0.5 g of the sample was dispersed in 40 mL of distilled water at 25 °C and agitated intermittently for 30 min, then centrifuged at 3000× *g* for 20 min. The collected supernatant was dried at 105 °C, and WSI was calculated as the dry weight of soluble solids relative to the initial dry sample weight. Steady-shear rheological behavior of RVA-derived pastes was analyzed using a controlled-stress rheometer (Discovery HR-10; TA Instruments, New Castle, DE, USA) equipped with a 40 mm parallel-plate geometry. Flow sweep tests were performed at 0.1–1000 s^−1^, and η_50_ was obtained at 50 s^−1^. Pasting properties were assessed using a Rapid Visco Analyzer (RVA4500, PerkinElmer, Shelton, CT, USA) following AACC International Method 61-02.01, with sample weights adjusted to a 12% moisture basis. Setback viscosity was calculated as the difference between the final and trough viscosities.

### 2.4. Experimental Design and Group Structure

All cross-validation procedures were performed at the 108-group level, with all three replicates within a given group assigned to the same fold to preserve the dependency structure of the dataset and prevent replicate leakage between the training and validation subsets [24]. Model performance metrics were calculated using replicate-level predictions, whereas the validation framework itself was defined at the 108-group level.

### 2.5. Machine Learning Framework

#### 2.5.1. Input Formulations

Three input formulations were evaluated. Model A included only process-related variables (slurry concentration, ultrasonication amplitude, and treatment time). Model B incorporated three cultivar-level composition descriptors in addition to the process variables, namely amylose, protein, and fiber contents. These were chosen as readily measured composition descriptors relevant to rice flour functionality [13,14]. Model C replaced these composition descriptors with cultivar identity encoded by using a one-hot representation. Because each cultivar corresponded to a distinct compositional profile, comparison between Model B and Model C enabled assessment of the extent to which the three composition descriptors could functionally substitute for cultivar identity within the current dataset. Each response variable—WSI, η_50_, and Setback—was modeled independently under all three input formulations. The input formulation is summarized in Table 1.

Cultivar labels in Model C were encoded as six binary indicators (one per cultivar), with no reference category dropped, yielding nine features in combination with three process variables. Repeated nested group CV = repeated nested group K-fold cross-validation (Section Repeated Nested Group K-Fold Cross-Validation). LOCO-CV = Leave-one-cultivar-out cross-validation (Section Leave-One-Cultivar-Out Cross-Validation).

#### 2.5.2. Algorithms

Five machine-learning algorithms, representing regularized linear, latent-variable, kernel-based, bagging, and boosting approaches, were evaluated: ElasticNet [25], partial least squares regression (PLS [26]), support vector regression with a radial basis function kernel (SVR [27]), random forest (RF; Breiman [28]), and extreme gradient boosting (XGBoost [29]). All models were implemented using scikit-learn pipeline frameworks, with predictor variables standardized using StandardScaler within each training fold. Model hyperparameters were optimized via grid search within the inner-group cross-validation loop (Section 2.5.3), and root mean squared error (RMSE) was used as the refitting criterion. The evaluated hyperparameter grids are summarized in Table S2.

#### 2.5.3. Cross-Validation Strategies

Two cross-validation protocols were used to evaluate two generalization scenarios. Within-domain prediction referred to model performance on unseen cultivar–process groups derived from cultivars represented in the training dataset, whereas unseen-cultivar transfer referred to performance on cultivars entirely excluded from model training. Within-domain performance was assessed using repeated nested group K-fold cross-validation, while transferability to unseen cultivars was evaluated using leave-one-cultivar-out cross-validation (LOCO-CV). Nested cross-validation was used to separate hyperparameter optimization from outer validation, thereby reducing model-selection bias under limited sample conditions [30,31,32]. As described in Section 2.4, all data splits were conducted at the group level.

##### Repeated Nested Group K-Fold Cross-Validation

This protocol served as the primary evaluation framework for within-domain prediction and was applied to all comparisons among Models A, B, and C. The outer validation loop consisted of group-based 5-fold cross-validation, repeated five times with different random shuffles, yielding 25 outer validation splits. Each outer split corresponds to an approximately 80%/20% group-level train–validation partition, and LOCO-CV used five cultivars for training and one held-out cultivar for validation. Within each outer split, model hyperparameters were optimized using inner group-based 5-fold cross-validation performed exclusively on the outer training groups. The optimal model was subsequently retrained using the full outer training dataset and evaluated on the corresponding outer validation groups. Predictive performance was summarized as the mean and standard deviation across all 25 outer splits.

##### Leave-One-Cultivar-Out Cross-Validation

Since cultivar identity cannot be assigned to cultivars absent from the training set, LOCO-CV was used to evaluate whether composition descriptors could function as transferable cultivar representations. Accordingly, this procedure was applied only to Models A and B. In each LOCO-CV iteration, all groups associated with one cultivar were held out for the outer validation set, whereas the remaining five cultivars were used for model training and inner-group-based 5-fold hyperparameter optimization. To quantify compositional dissimilarity between the held-out cultivar and the training cultivars, multivariate composition distance was calculated as the z-scaled Euclidean nearest-neighbor distance (z_NN) after standardizing each composition variable using the training dataset. Cultivars exhibiting z_NN values substantially exceeding those of the remaining held-out cultivars were interpreted as representing boundary extrapolation cases during cultivar-specific LOCO-CV evaluation.

#### 2.5.4. SHAP Analysis

SHAP values were calculated for XGBoost Model B using the TreeSHAP algorithm [21]. To maintain a leakage-free evaluation framework, feature attributions were constructed using a cross-fitted strategy that mirrored the repeated nested group cross-validation procedure. Specifically, for each of the 25 outer validation splits, XGBoost Model B was retrained on the corresponding outer training groups, using the hyperparameters selected for that split, and SHAP values were computed exclusively for observations in the outer validation groups. SHAP attributions from 25 splits were subsequently pooled, such that each observation contributed within multiple out-of-fold evaluation contexts under the repeated 5-fold × 5-repeat structure. Global feature importance was calculated as the mean absolute SHAP value of each feature across all pooled out-of-fold observations. Process contribution was calculated as the sum of mean absolute SHAP values for concentration, amplitude, and time. Composition contribution was the sum of amylose, protein, and fiber. Both contributions were normalized relative to the total mean absolute SHAP contribution.

### 2.6. Performance Comparison and Reproducibility

Model performance was evaluated using the coefficient of determination (R^2^), root mean square error (RMSE), and normalized RMSE (nRMSE), which were calculated as follows:

(1) $$R^{2}=1-\left[\sum_{i=1^{n}}{\left(y_{i}-{\hat{y}}_{i}\right)}^{2}/\sum_{i=1^{n}}{\left(y_{i}-\bar{y}\right)}^{2}\right],$$

(2) $$\mathrm{RMSE}=\sqrt{\left[\left(1/n\right)\sum_{i=1^{n}}{\left(y_{i}-{\hat{y}}_{i}\right)}^{2}\right],}$$

(3) nRMSE (%) = [RMSE/(y_max_ − y_min_)] × 100,

where y_i_ and ${\hat{y}}_{i}$ are the observed and predicted values, respectively; $\bar{y}$ is the mean of the observed values; n is the number of observations; and y_max_ and y_min_ are the maximum and minimum observed values of each target response across the entire dataset, respectively.

Pairwise differences in predictive performance among input formulations were assessed within the same algorithm, response variable, and outer validation split. Comparisons included A–B and B–C under repeated nested group cross-validation, and A–B under LOCO-CV. For each comparison, differences in R^2^, RMSE, and nRMSE were summarized together with the number of validation splits in which lower RMSE was achieved. As a descriptive reference for partitioning response variance between process variables and cultivar identity to response variation, each response variable was additionally modeled using ordinary least squares regression with process variables alone and with process variables plus cultivar identity.

Formal statistical hypothesis testing was not performed because repeated outer validation splits reused the same finite set of experimental groups and could not be considered statistically independent observations. Consequently, model comparisons were interpreted descriptively based on the magnitude and consistency of paired performance differences across splits. LOCO-CV results were likewise interpreted descriptively because of the limited number of held-out cultivar folds (n = 6 per algorithm × target combination). All analyses were conducted in Python 3.13 using scikit-learn 1.7, xgboost 3.2, shap 0.51, pandas 2.3, and numpy 2.3, with random_state = 42 applied to all stochastic procedures.

## 3. Results and Discussion

### 3.1. Cultivar-Level Variability in Response Variables

**Figure 1:**
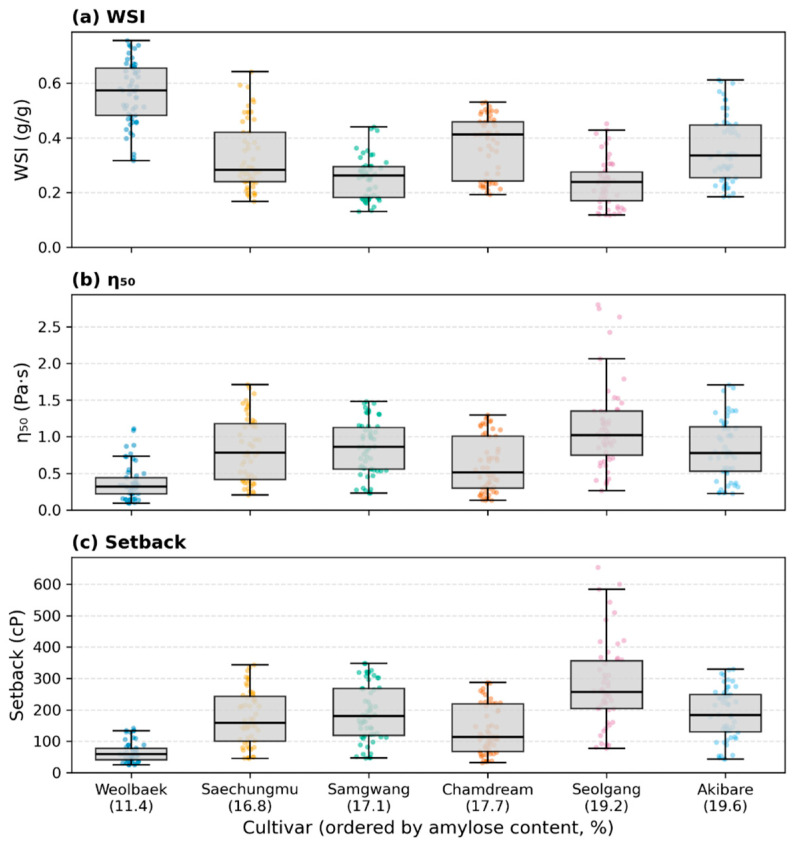
Distributions of (**a**) WSI (g/g), (**b**) η_50_ (Pa·s), and (**c**) Setback (cP) for six rice cultivars, ordered by amylose content (%). Each point is one ultrasonication condition × replicate.

All three responses exhibited cultivar-specific differences in distribution, with median values ranging from 0.238 to 0.574 for WSI, from 0.318 to 1.024 Pa·s for η_50_, and from 59 to 257 cP for Setback (Figure 1). Weolbaek (amylose 11.4%) exhibited the highest median WSI (0.574) and the lowest median η_50_ (0.318 Pa·s) and median Setback (59 cP); its amylose content was markedly lower than that of the other five cultivars (16.8–19.6%). Typically, low-amylose rice flour contains a large amylopectin fraction, which can enhance dispersion and limit post-cooling reassociation [33,34]. Protein and fiber did not account for this contrast: Weolbaek and Seolgang both contained high protein contents (6.81% and 7.18%, respectively) yet considerably different median responses (WSI: 0.574 vs. 0.238; η_50_: 0.318 vs. 1.024 Pa·s; and Setback: 59 vs. 257 cP, respectively), and Weolbaek and Chamdream contained the same fiber content (0.92%) but differed in median η_50_ (0.318 vs. 0.514 Pa·s) and Setback (59 vs. 115 cP).

Among the remaining five cultivars (16.8–19.6% amylose), the response medians did not follow a simple amylose-based order. Chamdream contained higher amylose content (17.7%) than Saechungmu (16.8%) and Samgwang (17.1%), but exhibited higher median WSI (0.413 vs. 0.283 and 0.261, respectively) and lower median η_50_ (0.514 vs. 0.781 and 0.859 Pa·s) and median Setback (115 vs. 159 and 181 cP, respectively). These observations indicate that no single composition descriptor explained the response medians.

### 3.2. Process and Composition Variables as Model Inputs

Model performance was evaluated across the three input formulations, five algorithms, and three response variables under repeated nested-group cross-validation; Table 2 reports the results for the best-performing algorithm (XGBoost), and the complete results for all five algorithms are provided in Table S3 (Supplementary Materials). The process-only model (Model A) exhibited limited predictive performance overall, with R^2^ values below 0.55 across all response–algorithm combinations: 0.067–0.246 for WSI, 0.236–0.441 for Setback, and 0.423–0.547 for η_50_. Performance also differed across the outer splits, with R^2^ standard deviations ranging from 0.12 to 0.26. The addition of amylose, protein, and fiber (Model B) improved the prediction for every response–algorithm combination. For XGBoost, Model B achieved R^2^ values of 0.81 ± 0.10 for WSI, 0.83 ± 0.06 for η_50_, and 0.90 ± 0.04 for Setback. For the same responses, the nRMSE decreased from 20.4%, 11.7%, and 13.2% in Model A to 9.7%, 6.8%, and 5.1% in Model B, respectively. The same direction of improvement was found for the other four algorithms, and the outer-split variation was typically smaller for Model B than for Model A (R^2^ standard deviations of 0.04–0.19 vs. 0.12–0.26). The largest gain was observed for WSI. Setback also exhibited distinct improvement, with RMSE decreasing from approximately 79–91 cP under Model A to 32–52 cP under Model B. For η_50_, Model A baseline performance was higher, but Model B still increased R^2^ to 0.76–0.83 across algorithms. In the auxiliary linear regression, adding cultivar identity as a process variable increased the adjusted R^2^ by 0.24–0.52 (Table S4), suggesting that Model A’s performance was limited by the explanatory range of the process variables.

**Figure 2:**
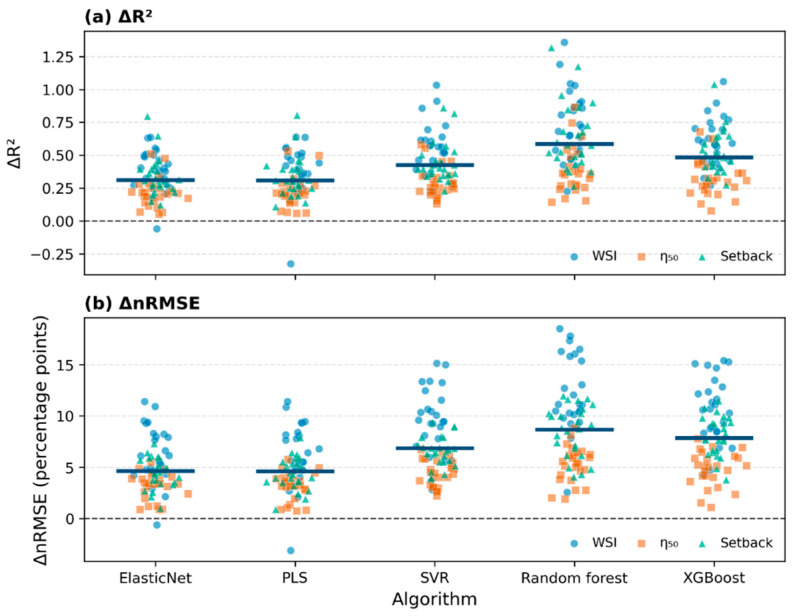
Gain of Model B over Model A across algorithms and responses. (**a**) ΔR^2^ = R^2^(B) − R^2^(A); (**b**) nRMSE reduction = nRMSE(A) − nRMSE(B), in percentage points. Positive values favor Model B; bars are within-algorithm means.

**Figure 3:**
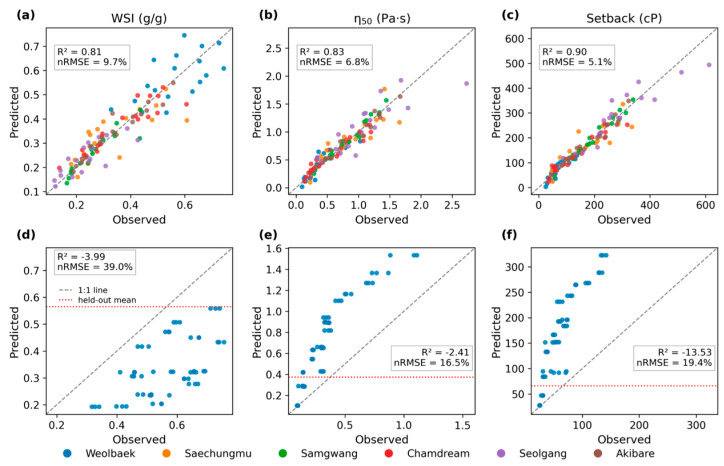
Observed versus predicted values for XGBoost Model B. (**a**–**c**) Within-domain predictions for (**a**) WSI, (**b**) η_50_, and (**c**) Setback, as out-of-fold predictions from the repeated nested group cross-validation, averaged within each cultivar–process group (108 groups); color denotes cultivar. (**d**–**f**) Leave-one-cultivar-out predictions for the worst-performing held-out cultivar, Weolbaek, for (**d**) WSI, (**e**) η_50_, and (**f**) Setback; the red dotted line indicates the mean of the held-out cultivar responses.

Figure 2 shows that the advantage of Model B was also consistent at the outer-split level. Across the 375 paired comparisons (15 combinations × 25 splits), Model B exhibited a higher R^2^ and lower nRMSE than Model A in 373 cases (99.5%), with two exceptions occurring for WSI under PLS or ElasticNet. Mean ΔR^2^ values were 0.53 for WSI, 0.29 for η_50_, and 0.45 for Setback. The mean nRMSE reductions were 9.0, 4.2, and 6.4 percentage points, respectively. The same comparisons held when metrics were computed at the 108-group level rather than at the replicate level (Table S5). The advantage of adding material descriptors to process inputs has been well established in other process modeling contexts [35,36]. However, adding less relevant predictors may not affect the performance or even hamper it [37,38]. In the present study, amylose, protein, and fiber added predictive information that was not available from the treatment conditions alone. Observed-versus-predicted plots for XGBoost Model B showed predictions distributed around the 1:1 line for all three responses, without marked systematic bias (Figure 3). The advantage of Model B over Model A was robust to the error-normalization choice (Table S3): training-range-normalized nRMSE was within approximately 0.3 percentage points of the range-normalized values, and CV-RMSE relative to the response mean showed the same improvement.

### 3.3. Composition-Descriptor Versus Cultivar-Label Inputs

**Figure 4:**
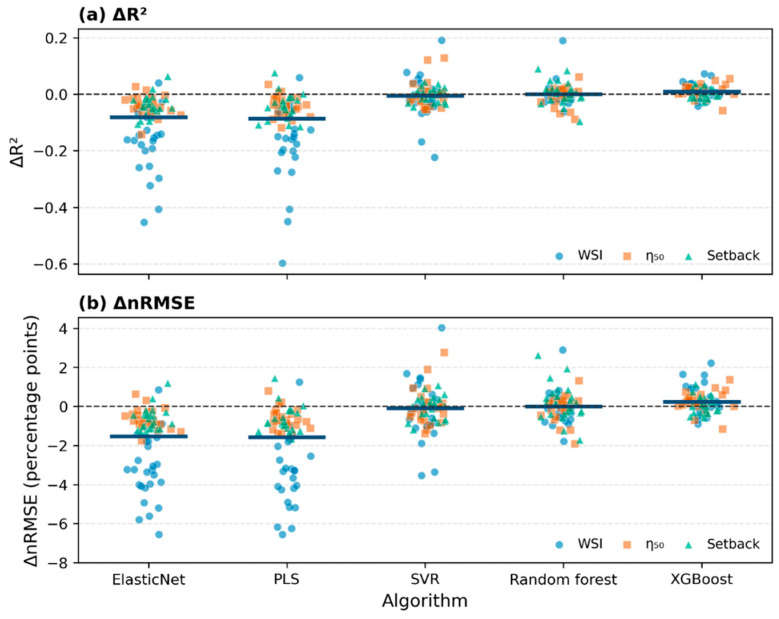
Model B (process + composition) vs. Model C (process + cultivar one-hot). (**a**) ΔR^2^ = R^2^(B) − R^2^(C); (**b**) nRMSE reduction = nRMSE(C) − nRMSE(B), in percentage points. Positive values favor Model B; bars are within-algorithm means.

Models B and C were compared to evaluate the two cultivar representations (continuous composition descriptors and cultivar labels). The pairwise comparison results, summarized in Table 2 and presented in Figure 4, were strongly dependent on the algorithm class. Under nonlinear algorithms, Models B and C achieved comparable performances. Mean ΔR^2^ (B − C) was −0.004 for SVR, near-zero for RF, and +0.009 for XGBoost across all targets and outer splits, and remained within ±0.02 across all algorithm × target combinations.

The two linear algorithms showed a greater advantage for Model C, particularly for WSI, likely reflecting the encoding itself. Cultivar labels give a direct way for the linear model to represent cultivar-specific shifts, whereas Model B had to express cultivar differences through linear combinations of amylose, protein, and fiber [39]. This result is consistent with that in Section 3.1, where the response patterns were not captured by a simple ordering of amylose, protein, or fiber.

The composition descriptors approximately matched the predictive performance of the cultivar labels under nonlinear algorithms. The substitution of cultivar labels with continuous composition descriptors has been used to model variety-related variations in rice [40]. This close performance should not be interpreted as evidence of independent compositional effects, because each cultivar had a fixed compositional profile, and predictive performance does not by itself imply a causal contribution [41]. A stricter test of cultivar transfer is whether these descriptors can predict cultivars that are not represented in the training.

### 3.4. Leave-One-Cultivar-Out Validation

**Figure 5:**
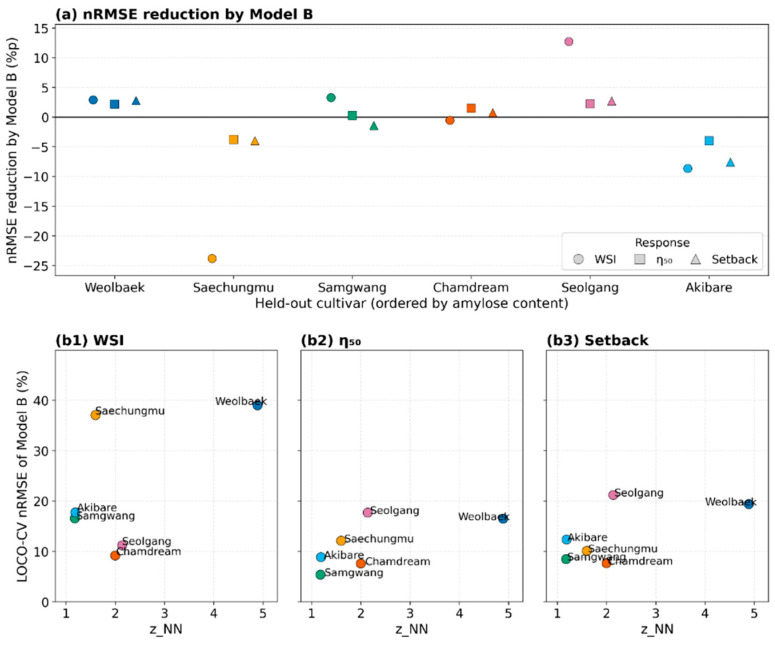
LOCO-CV of XGBoost Models A and B. (**a**) nRMSE reduction = nRMSE(A) − nRMSE(B), in percentage points; cultivars ordered by amylose content. (**b1**–**b3**) LOCO-CV nRMSE of Model B vs. z_NN.

LOCO-CV provided a diagnostic test to determine whether the composition descriptors could support the prediction of a cultivar that was absent from training. Observed-versus-predicted plots for the worst-performing held-out cultivar (Weolbaek) are shown in Figure 3d–f. The XGBoost results for Models A and B are summarized in Table 3 and Figure 5. The high repeated CV performance of Model B was not transferred uniformly to the held-out cultivars, and performance varied substantially across the six folds. The highest failure occurred when Weolbaek was held out, which produced Model B R^2^ values of −3.985 for WSI, −2.406 for η_50_, and −13.530 for Setback. Negative R^2^ values indicate that, for a given held-out cultivar, the LOCO-CV predictions were less accurate than a baseline defined by the mean of the observed responses in that held-out fold. Weolbaek exhibited the largest z_NN (4.89) from the training set, which was considerably above the range for other cultivars (1.18–2.14). A sensitivity LOCO-CV after excluding Weolbaek increased the mean Model B R^2^ from 0.09 to 0.64 for η_50_ and from −1.95 to 0.52 for Setback, while WSI improved from −1.08 to 0.08 but remained difficult to predict (Table S6).

The LOCO performance varied considerably among the other five cultivars (Figure 5). Model B reduced nRMSE relative to Model A for Seolgang (notably WSI, a ~12.7%p reduction) and for Chamdream (η_50_ and Setback)—the two cultivars with the largest composition distances among the non-Weolbaek folds (z_NN = 2.14 and 2.00). By contrast, Saechungmu and Akibare, which had smaller distances (z_NN = 1.60 and 1.19), showed increased nRMSE across all three responses; Saechungmu, although within the training range of each composition variable, showed the most marked WSI degradation (nRMSE rose to ~37%). Among the non-Weolbaek cultivars, composition distance did not by itself determine whether transfer improved or degraded, consistent with the limited extrapolation of tree-based models beyond the training input range [42] and with the bias that can arise when a held-out group differs systematically from the training set [43]. The degradation of the closer cultivars instead points to cultivar-level traits not captured by the three descriptors, such as amylopectin chain-length distribution [44], starch fine structure [45], and protein subunit composition [46]. Thus, the composition-descriptor model was useful for within-domain prediction, but its transfer to the held-out cultivars was inconsistent.

### 3.5. SHAP-Based Feature Attribution

**Figure 6:**
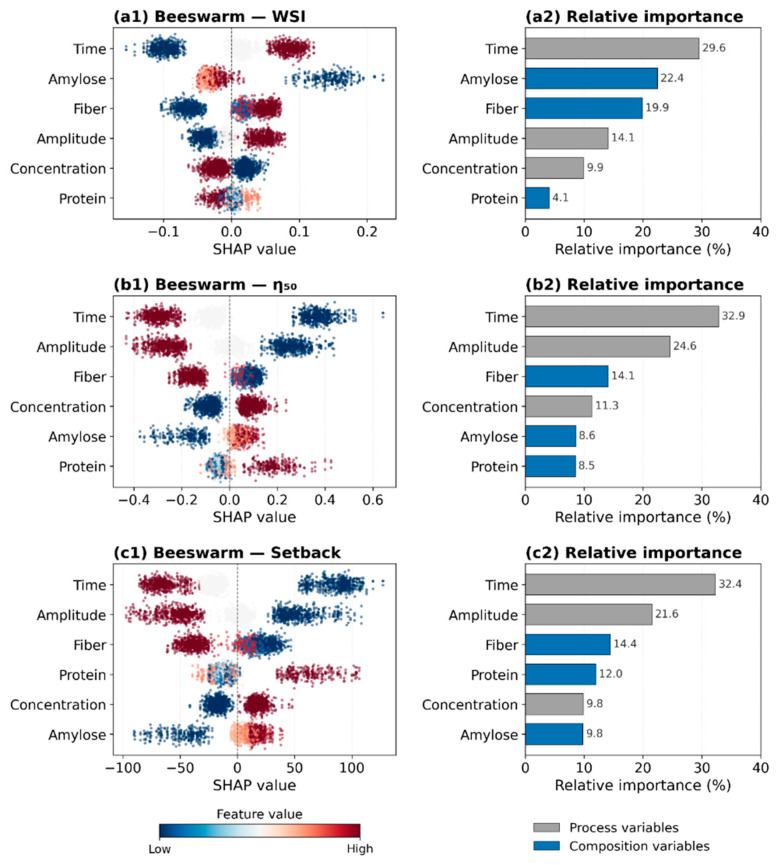
SHAP analysis of XGBoost Model B. (**a1**,**b1**,**c1**) Beeswarm plots; color indicates feature value. (**a2**,**b2**,**c2**) Relative importance.

XGBoost was selected for the SHAP analysis because it consistently achieved high Model B performance across the three responses (Section 3.2; Table 2). The cross-fitted SHAP results for within-domain XGBoost Model B are summarized in Figure 6 and Table S7. The beeswarm plots show signed SHAP distributions, whereas the bar plots show the relative importance calculated from the normalized mean absolute SHAP values. Among the process variables, time had the largest single attribution for all three responses, accounting for 29.6%, 32.9%, and 32.4% of total mean absolute SHAP values for WSI, η_50_, and Setback, respectively. Longer treatment time shifted predicted WSI upward and predicted η_50_ and Setback downward. Amplitude showed the same direction: higher amplitude increased predicted WSI but decreased predicted η_50_ and Setback. These results are consistent with the expected effects of intensified ultrasonication on starch dispersion, viscosity reduction, and post-cooling reassociation [8,10]. Concentration had a smaller attribution than time and amplitude, with higher concentration decreasing predicted WSI and increasing predicted η_50_ and Setback.

The composition descriptors also received substantial SHAP attributions; however, the patterns differed across responses. For WSI, amylose and fiber were the main composition variables, contributing 22.4% and 19.9%, respectively. Rice flour containing low amylose content typically exhibits increased swelling and dispersion, which is consistent with the positive SHAP values of low amylose content in the WSI prediction [45]. For η_50_, fiber was the largest composition contributor; however, its relative importance (14.1%) was lower than those of Time and Amplitude. Higher fiber values were associated with lower predicted η_50_ and Setback, consistent with previous reports that bran hemicellulose fractions decrease the viscosity of rice flour pastes [47]. For Setback, fiber, protein, and amylose contributed at comparable levels among the composition variables, with relative importance values of 14.4%, 12.0%, and 9.8%, respectively. For Setback, high amylose values tended to lie on the positive side of the SHAP distribution, consistent with the role of starch chain reassociation during cooling [34,48]. Higher protein values were also associated with positive SHAP contributions to Setback, which is consistent with previous findings that protein–starch interactions in rice systems can influence retrogradation and setback behavior [49].

The partition between process-group and composition-group SHAP magnitudes was approximately 54%/46% for WSI, 69%/31% for η_50_, and 64%/36% for Setback (Figure 6). Process variables were modestly dominant overall, whereas composition descriptors accounted for a substantial share, particularly for WSI. These attributions describe the fitted within-domain model rather than the independent effects across unconstrained composition combinations. Because each cultivar had a fixed composition profile and the descriptors are correlated with cultivar identity and with unmeasured cultivar-specific traits, these SHAP values should be interpreted as model-based feature attributions rather than as evidence of independent physicochemical effects of amylose, protein, or fiber.

## 4. Conclusions

In the present study, amylose, protein, and fiber contents were examined as composition descriptors for predicting flow-cell ultrasonication-induced changes in pregelatinized rice flour across six Korean Japonica rice cultivars and three response variables (WSI, η_50_, and Setback). Incorporation of these descriptors considerably improved within-domain predictive performance relative to process-only baseline and produced performance comparable to that achieved with cultivar labels under nonlinear algorithms. However, LOCO-CV showed inconsistent transfer of the composition-descriptor model across the six held-out cultivars. The largest prediction failure occurred for the cultivar at the greatest multivariate compositional distance from the training set (Weolbaek, z_NN = 4.89), well beyond the range observed for the remaining five cultivars (1.18–2.14). Even within this compositional range, predictive transferability across cultivars remained inconsistent. Because only six cultivars were tested, these findings are specific to this dataset, and the transferability of the composition-descriptor model to other cultivars therefore remains uncertain. These findings indicate that multivariate distance to the training set can serve as a useful indicator of clear extrapolation cases. However, it does not guarantee reliable transfer for cultivars located within the apparent training-domain composition space. Altogether, the results distinguish between-domain prediction from unseen-cultivar transfer as separate evaluation regimes when composition descriptors are used in place of cultivar identity. Within the validated cultivar–process domain, the composition-descriptor approach offers a route for preliminary screening of ultrasonication outcomes from compositional data, which can help prioritize cultivar–process combinations for further formulation testing in plant-based beverages and convenience foods. Achieving reliable extrapolation to compositionally distinct cultivars will likely require both broader cultivar representation in the training dataset and expansion of the descriptor space to include additional structural and compositional variables, including amylopectin chain-length distribution, damaged starch content, starch granule size, and protein fraction composition. More broadly, this within-domain versus unseen-cultivar distinction is methodologically relevant when continuous material descriptors substitute for categorical source identifiers in heterogeneous biological and food-processing systems; group-based validation paired with a multivariate distance diagnostic such as z_NN can support identification of candidate extrapolation cases.

## Figures and Tables

**Table 1:** Three input formulations compared in this study.

Model	Process Variables	Cultivar Information		Total Features	Used in CV Protocols
		Composition Descriptors	Cultivar Labels		
A (process only)	Concentration, Amplitude, Time	-	-	3	Standard nested CV; LOCO-CV
B (process + composition descriptors)	Concentration, Amplitude, Time	Amylose, Protein, Fiber	-	6	Standard nested CV; LOCO-CV
C (process + cultivar labels)	Concentration, Amplitude, Time	-	Cultivar labels (six-dimensional one-hot encoding)	9	Standard nested CV only

**Table 2:** Predictive performance of Models A, B, and C across response variables for the XGBoost model under repeated nested group cross-validation.

Response	Model	R^2^	RMSE	nRMSE (%)
WSI	A	0.203 ± 0.209	0.130 ± 0.020	20.41 ± 3.12
	B	0.807 ± 0.097	0.062 ± 0.015	9.71 ± 2.29
	C	0.798 ± 0.090	0.064 ± 0.013	10.04 ± 2.10
η_50_	A	0.514 ± 0.127	0.316 ± 0.058	11.66 ± 2.15
	B	0.833 ± 0.056	0.185 ± 0.044	6.84 ± 1.61
	C	0.820 ± 0.060	0.193 ± 0.047	7.10 ± 1.73
Setback	A	0.373 ± 0.166	83.045 ± 15.301	13.20 ± 2.43
	B	0.904 ± 0.040	32.300 ± 7.900	5.14 ± 1.26
	C	0.900 ± 0.041	32.957 ± 7.407	5.24 ± 1.18

Note. Values are means ± standard deviations across 25 outer validation splits. The model definitions are listed in Table 1. RMSE units: dimensionless for WSI, Pa·s for η_50_, cP for Setback. R^2^, coefficient of determination; RMSE, root mean square error; nRMSE, normalized RMSE expressed as a percentage of the response range.

**Table 3:** Leave-one-cultivar-out validation performance of Models A and B for each held-out cultivar (XGBoost).

Held-Out Cultivar	Response	Model A R^2^	Model B R^2^	ΔR^2^	z_NN
Weolbaek	WSI	−4.755	−3.985	0.77	4.89
	η_50_	−3.364	−2.406	0.959	
	Setback	−17.985	−13.53	4.456	
Saechungmu	WSI	0.539	−2.604	−3.143	1.60
	η_50_	0.73	0.431	−0.299	
	Setback	0.802	0.461	−0.341	
Samgwang	WSI	−1.877	−1.005	0.872	1.18
	η_50_	0.817	0.834	0.017	
	Setback	0.755	0.647	−0.108	
Chamdream	WSI	0.743	0.708	−0.035	2.00
	η_50_	0.579	0.705	0.126	
	Setback	0.547	0.622	0.075	
Seolgang	WSI	−2.257	0.286	2.543	2.14
	η_50_	0.109	0.297	0.187	
	Setback	−0.235	0.03	0.266	
Akibare	WSI	0.769	0.119	−0.65	1.19
	η_50_	0.896	0.652	−0.244	
	Setback	0.865	0.092	−0.773	

Note. The model definitions are listed in Table 1. ΔR^2^ was calculated as R^2^ (Model B) − R^2^ (Model A); positive values indicate improvement under Model B.

## Data Availability

Supplementary Data include the analysis-ready input dataset used for the machine-learning analyses, model performance summaries, pairwise comparison results, selected hyperparameters, and out-of-fold prediction outputs. The raw experimental data supporting the findings of this study are available from the corresponding author upon reasonable request.

## Supplementary Materials

The following supporting information can be downloaded at: https://www.mdpi.com/article/10.3390/foods15132268/s1, Table S1. Cultivar-level composition profiles and descriptive statistics of ultrasonication response variables; Table S2. Hyperparameter grids used for inner-loop grid search across five machine learning algorithms; Table S3. Full predictive performance of all five algorithms across response variables and input formulations, including training-range-normalized nRMSE and mean-relative CV-RMSE; Table S4. Process-only and process-plus-cultivar linear regression for each response variable; Table S5. Mean cross-validation performance computed at the replicate level versus at the cultivar–process group-mean level; Table S6. Leave-one-cultivar-out validation performance after excluding Weolbaek (XGBoost); Table S7. Cross-fitted SHAP feature importance for the within-domain XGBoost Model B; Dataset S1. ready to analysis data for ML analysis.

## Abbreviations

The following abbreviations are used in this manuscript:

SHAP	Shapley Additive exPlanations
WSI	Water solubility index
PLS	Partial least squares
SVR	Support vector regression
RMSE	Root mean squared error
LOCO-CV	Leave-one-cultivar-out cross-validation
Z_NN	Z-scaled Euclidean nearest-neighbor distance
