# Supplementary material for: Composition Descriptors and Cultivar Transferability in Machine-Learning Models of Ultrasonication-Induced Functional Properties of Rice Flour
Source: Foods. 2026 Jun 24;15(13):2268. doi: 10.3390/foods15132268 (PMC13361452; doi:10.3390/foods15132268)
Supplement: Supplementary file 1 [file foods-15-02268-s001.zip › Table_S1_composition_and_response.pdf]

Table S1. Cultivar-level composition profiles and descriptive statistics of ultrasonication response variables.

Cultivar	Amylose (%)	Protein (%)	Fiber (%)	Response	Mean	SD	Median	Q1	Q3	IQR	Min	Max
Weolbaek	11.37	6.81	0.92	WSI (g/g)	0.566	0.113	0.574	0.482	0.656	0.174	0.317	0.756
				η_{50} (Pa·s)	0.375	0.245	0.318	0.221	0.44	0.219	0.092	1.112
				Setback (cP)	66.148	32.295	59	40.5	77.75	37.25	25	142
Saechungmu	16.8	6.54	0.84	WSI (g/g)	0.332	0.126	0.283	0.24	0.421	0.18	0.167	0.643
				η_{50} (Pa·s)	0.834	0.439	0.781	0.413	1.177	0.764	0.204	1.71
				Setback (cP)	174.259	87.26	159	100	243.25	143.25	46	344
Samgwang	17.14	5.85	0.25	WSI (g/g)	0.254	0.075	0.261	0.183	0.294	0.112	0.13	0.441
				η_{50} (Pa·s)	0.848	0.361	0.859	0.559	1.127	0.568	0.229	1.481
				Setback (cP)	189.907	90.423	180.5	118.25	268	149.75	47	348
Chamdream	17.73	5.65	0.92	WSI (g/g)	0.373	0.109	0.413	0.243	0.458	0.216	0.192	0.531
				η_{50} (Pa·s)	0.616	0.382	0.514	0.297	1.007	0.71	0.13	1.298
				Setback (cP)	136.593	79.047	114.5	67.5	219	151.5	32	288
Seolgang	19.23	7.18	0.26	WSI (g/g)	0.236	0.085	0.238	0.171	0.275	0.105	0.117	0.453
				η_{50} (Pa·s)	1.122	0.578	1.024	0.747	1.348	0.601	0.266	2.804
				Setback (cP)	278.019	136.616	256.5	204	356.25	152.25	78	654
Akibare	19.59	6.27	0.31	WSI (g/g)	0.359	0.122	0.335	0.254	0.448	0.193	0.185	0.613
				η_{50} (Pa·s)	0.818	0.411	0.778	0.526	1.133	0.607	0.222	1.707
				Setback (cP)	188.074	82.239	183.5	130.25	248.75	118.5	43	330

Note. Composition values represent the proximate composition of each cultivar used as model inputs. Descriptive statistics summarize the distribution of each response variable across all ultrasonication conditions, corresponding to the boxplots in Figure 1. WSI, water solubility index; η_{50}, apparent viscosity at 50 s^{-1}; Setback, setback viscosity; SD, standard deviation; Q1, first quartile; Q3, third quartile; IQR, interquartile range.
