# Supplementary material for: Composition Descriptors and Cultivar Transferability in Machine-Learning Models of Ultrasonication-Induced Functional Properties of Rice Flour
Source: Foods. 2026 Jun 24;15(13):2268. doi: 10.3390/foods15132268 (PMC13361452; doi:10.3390/foods15132268)
Supplement: Supplementary file 1 [file foods-15-02268-s001.zip › Table_S2_hyperparameter_grid.pdf]

Table S2. Hyperparameter grids used for inner-loop grid search across five machine learning algorithms.

Algorithm	Hyperparameter	Values searched
**ElasticNet**	α	0.001, 0.01, 0.1, 1
	l1_ratio	0.1, 0.3, 0.5, 0.7
**PLS**	n_components	1, 2, …, min (n_features, 5)
**SVR (RBF kernel)**	C	1, 10, 100
	ε	0.01, 0.05, 0.1
	γ	scale, 0.01, 0.1
**Random Forest**	n_estimators	200 (fixed)
	max_depth	None, 5
	min_samples_leaf	1, 3
	max_features	sqrt, 1.0
**XGBoost**	n_estimators	100, 300
	max_depth	2, 3
	learning_rate	0.05, 0.1
	subsample	0.8, 1.0
	colsample_bytree	0.8, 1.0
	reg_lambda	1, 5

Note. All other hyperparameters were left at scikit-learn (v1.7) and xgboost (v3.2) defaults. PLS n_components ranged from 1 to min (n_features, 5): 3 for Model A, 5 for Model B (capped from 6), and 5 for Model C (capped from 9). The same grids were used for both repeated nested group CV and LOCO-CV. Inner-loop grid search was performed independently within each outer fold to prevent information leakage.
