# Supplementary material for: Composition Descriptors and Cultivar Transferability in Machine-Learning Models of Ultrasonication-Induced Functional Properties of Rice Flour
Source: Foods. 2026 Jun 24;15(13):2268. doi: 10.3390/foods15132268 (PMC13361452; doi:10.3390/foods15132268)
Supplement: Supplementary file 1 [file foods-15-02268-s001.zip › Table_S3_Full_performance_alt_metrics.pdf]

Table S3. Full predictive performance with alternative error normalizations.

Response	Algorithm	Model	R^2	RMSE	nRMSE (range, %)	nRMSE (train, %)	CV-RMSE (mean, %)
WSI	ElasticNet	A	0.245 ± 0.208	0.127 ± 0.019	19.83	19.95	35.87
		B	0.627 ± 0.169	0.086 ± 0.013	13.50	13.58	24.43
		C	0.792 ± 0.079	0.065 ± 0.011	10.21	10.28	18.48
	PLS	A	0.246 ± 0.210	0.127 ± 0.019	19.80	19.93	35.83
		B	0.618 ± 0.190	0.087 ± 0.012	13.58	13.66	24.57
		C	0.792 ± 0.081	0.065 ± 0.011	10.20	10.27	18.46
	SVR	A	0.227 ± 0.210	0.129 ± 0.022	20.11	20.24	36.39
		B	0.777 ± 0.095	0.068 ± 0.012	10.57	10.64	19.13
		C	0.788 ± 0.095	0.066 ± 0.016	10.33	10.39	18.70
	Random forest	A	0.067 ± 0.261	0.141 ± 0.024	22.05	22.18	39.90
		B	0.806 ± 0.080	0.063 ± 0.012	9.86	9.92	17.84
		C	0.805 ± 0.104	0.062 ± 0.013	9.77	9.84	17.68
	XGBoost	A	0.203 ± 0.209	0.130 ± 0.020	20.41	20.53	36.92
		B	0.807 ± 0.097	0.062 ± 0.015	9.71	9.77	17.57
		C	0.798 ± 0.090	0.064 ± 0.013	10.04	10.11	18.17
η_{50}	ElasticNet	A	0.547 ± 0.123	0.306 ± 0.061	11.27	11.76	39.74
		B	0.763 ± 0.069	0.221 ± 0.050	8.14	8.51	28.73
		C	0.803 ± 0.053	0.203 ± 0.052	7.48	7.84	26.40
	PLS	A	0.545 ± 0.125	0.306 ± 0.060	11.29	11.78	39.82
		B	0.761 ± 0.070	0.221 ± 0.050	8.16	8.53	28.79
		C	0.802 ± 0.053	0.203 ± 0.049	7.50	7.84	26.44
	SVR	A	0.541 ± 0.118	0.308 ± 0.059	11.34	11.83	40.01
		B	0.832 ± 0.037	0.188 ± 0.044	6.92	7.24	24.41
		C	0.833 ± 0.056	0.187 ± 0.056	6.90	7.24	24.35
	Random forest	A	0.423 ± 0.173	0.343 ± 0.061	12.64	13.16	44.59
		B	0.807 ± 0.064	0.200 ± 0.057	7.37	7.73	26.01
		C	0.813 ± 0.061	0.198 ± 0.058	7.29	7.64	25.72
	XGBoost	A	0.514 ± 0.127	0.316 ± 0.058	11.66	12.16	41.13
		B	0.833 ± 0.056	0.185 ± 0.044	6.84	7.13	24.13
		C	0.820 ± 0.060	0.193 ± 0.047	7.10	7.40	25.04
Setback	ElasticNet	A	0.421 ± 0.163	79.766 ± 14.647	12.68	13.37	46.33
		B	0.756 ± 0.065	51.724 ± 9.488	8.22	8.69	30.04
		C	0.794 ± 0.051	47.775 ± 9.696	7.60	8.04	27.75

PLS	A	0.415 ± 0.171	80.014 ± 14.501	12.72	13.40	46.47
	B	0.750 ± 0.072	52.115 ± 8.783	8.29	8.75	30.27
	C	0.793 ± 0.051	47.777 ± 8.890	7.60	8.04	27.75
SVR	A	0.441 ± 0.151	78.741 ± 16.060	12.52	13.21	45.74
	B	0.879 ± 0.041	37.053 ± 11.434	5.89	6.27	21.52
	C	0.880 ± 0.033	36.871 ± 10.142	5.86	6.22	21.42
Random forest	A	0.236 ± 0.247	91.120 ± 16.908	14.49	15.26	52.93
	B	0.871 ± 0.054	37.289 ± 9.500	5.93	6.27	21.66
	C	0.866 ± 0.048	38.501 ± 10.390	6.12	6.50	22.36
XGBoost	A	0.373 ± 0.166	83.045 ± 15.301	13.20	13.91	48.23
	B	0.904 ± 0.040	32.300 ± 7.900	5.14	5.43	18.76
	C	0.900 ± 0.041	32.957 ± 7.407	5.24	5.54	19.14

Note. Predictive performance of all five algorithms across the three response variables and three input formulations (Models A, B, C) under repeated nested group cross-validation (means ± SD across 25 outer splits). nRMSE (range) normalizes RMSE by the full-dataset response range; nRMSE (train) by the training-set range within each split; CV-RMSE (mean) is RMSE relative to the response mean. Model definitions follow Table 1.
