# Supplementary material for: Composition Descriptors and Cultivar Transferability in Machine-Learning Models of Ultrasonication-Induced Functional Properties of Rice Flour
Source: Foods. 2026 Jun 24;15(13):2268. doi: 10.3390/foods15132268 (PMC13361452; doi:10.3390/foods15132268)
Supplement: Supplementary file 1 [file foods-15-02268-s001.zip › Table_S4_Process_adjusted_cultivar.pdf]

Table S4. Process-only and process-plus-cultivar linear regression for each response variable.

Response	n	Adj. R^2 (process only)	Adj. R^2 (process + cultivar)	ΔAdj. R^2	Partial F (cultivar)	df (cultivar)	df (residual)
**WSI**	324	0.3307	0.8469	+0.5162	214.81	5	312
$\boldsymbol{\eta_{50}}$	324	0.5994	0.8394	+0.2400	95.71	5	312
**Setback**	324	0.4929	0.8344	+0.3415	131.78	5	312

Note. Linear regression of each response on (i) the three process variables (concentration, amplitude, time) and (ii) the same process variables plus cultivar identity (one-hot encoded, Weolbaek as reference). All 324 replicate observations were used. The partial F statistic compares the two nested models and is reported descriptively rather than as a hypothesis test, given that replicates within a cultivar–process group are not independent. Across all three responses, adding cultivar identity increased adjusted R^2 by 0.24–0.52 over the process-only model, indicating that the limited Model A performance reported in Section 3.2 reflects cultivar-associated variation not captured by process variables alone. WSI, water solubility index; η_{50}, apparent viscosity at 50 s^{-1}; Setback, setback viscosity.
