# Supplementary material for: Composition Descriptors and Cultivar Transferability in Machine-Learning Models of Ultrasonication-Induced Functional Properties of Rice Flour
Source: Foods. 2026 Jun 24;15(13):2268. doi: 10.3390/foods15132268 (PMC13361452; doi:10.3390/foods15132268)
Supplement: Supplementary file 1 [file foods-15-02268-s001.zip › Table_S5_GroupMean_vs_Replicate.pdf]

Table S5. Mean cross-validation performance computed at the replicate level versus at the cultivar–process group-mean level.

Response	Input formulation	Algorithm	R^2 (replicate)	R^2 (group-mean)	ΔR^2	RMSE (replicate)	RMSE (group-mean)	ΔRMSE
**WSI**	Model A	ElasticNet	0.245	0.246	+0.0013	0.1267	0.1262	-0.00050
		PLS	0.246	0.247	+0.0013	0.1265	0.1260	-0.00050
		SVR	0.227	0.228	+0.0013	0.1285	0.1280	-0.00049
		Random Forest	0.067	0.068	+0.0003	0.1409	0.1404	-0.00045
		XGBoost	0.203	0.204	+0.0011	0.1304	0.1299	-0.00049
	Model B	ElasticNet	0.627	0.631	+0.0035	0.0863	0.0855	-0.00073
		PLS	0.618	0.622	+0.0034	0.0868	0.0860	-0.00073
		SVR	0.777	0.781	+0.0045	0.0676	0.0666	-0.00093
		Random Forest	0.806	0.811	+0.0047	0.0630	0.0620	-0.00101
		XGBoost	0.807	0.812	+0.0048	0.0621	0.0610	-0.00105
	Model C	ElasticNet	0.792	0.797	+0.0047	0.0653	0.0643	-0.00097
		PLS	0.792	0.797	+0.0047	0.0652	0.0642	-0.00097
		SVR	0.788	0.793	+0.0047	0.0660	0.0651	-0.00098
		Random Forest	0.805	0.809	+0.0047	0.0625	0.0614	-0.00102
		XGBoost	0.798	0.803	+0.0047	0.0642	0.0632	-0.00100
η_{50}	Model A	ElasticNet	0.547	0.563	+0.0158	0.3055	0.2960	-0.00954
		PLS	0.545	0.561	+0.0157	0.3062	0.2966	-0.00953
		SVR	0.541	0.557	+0.0157	0.3076	0.2981	-0.00951
		Random Forest	0.423	0.435	+0.0121	0.3428	0.3344	-0.00842
		XGBoost	0.514	0.529	+0.0149	0.3162	0.3070	-0.00920
	Model B	ElasticNet	0.763	0.784	+0.0218	0.2209	0.2073	-0.01355
		PLS	0.761	0.783	+0.0217	0.2213	0.2079	-0.01337
		SVR	0.832	0.856	+0.0240	0.1877	0.1715	-0.01620
		Random Forest	0.807	0.830	+0.0229	0.2000	0.1853	-0.01470
		XGBoost	0.833	0.856	+0.0233	0.1855	0.1701	-0.01537
	Model C	ElasticNet	0.803	0.826	+0.0231	0.2029	0.1880	-0.01491
		PLS	0.802	0.825	+0.0230	0.2033	0.1885	-0.01481
		SVR	0.833	0.857	+0.0241	0.1872	0.1707	-0.01647
		Random Forest	0.813	0.836	+0.0229	0.1977	0.1830	-0.01469
		XGBoost	0.820	0.843	+0.0227	0.1925	0.1780	-0.01455
**Setback**	Model A	ElasticNet	0.421	0.424	+0.0035	79.77	79.21	-0.557
		PLS	0.415	0.418	+0.0034	80.01	79.46	-0.555
		SVR	0.441	0.444	+0.0037	78.74	78.17	-0.567
		Random Forest	0.236	0.238	+0.0018	91.12	90.63	-0.488
		XGBoost	0.373	0.376	+0.0030	83.04	82.51	-0.534
	Model B	ElasticNet	0.756	0.762	+0.0061	51.72	50.88	-0.846
		PLS	0.750	0.756	+0.0060	52.11	51.28	-0.836
		SVR	0.879	0.886	+0.0073	37.05	35.79	-1.268
		Random Forest	0.871	0.879	+0.0071	37.29	36.05	-1.237
		XGBoost	0.904	0.911	+0.0074	32.30	30.85	-1.448
	Model C	ElasticNet	0.794	0.801	+0.0065	47.78	46.85	-0.923
		PLS	0.793	0.799	+0.0064	47.78	46.86	-0.917
		SVR	0.880	0.887	+0.0073	36.87	35.63	-1.244
		Random Forest	0.866	0.873	+0.0071	38.50	37.29	-1.207
		XGBoost	0.900	0.907	+0.0074	32.96	31.57	-1.391

Note. Each row reports means across 25 outer cross-validation splits (group-based 5-fold × 5 repeats). Replicate-level metrics are computed from individual replicate predictions; group-mean metrics are computed after averaging the three replicates within each cultivar–process group. Both produce the same direction of comparison among input formulations and algorithms; group-mean metrics are slightly higher in R^2 and slightly lower in RMSE due to within-group averaging. WSI, water solubility index; η_{50}, apparent viscosity at 50 s^{-1}; Setback, setback viscosity; ΔR^2 and ΔRMSE, group-mean minus replicate-level.
