# Supplementary material for: Composition Descriptors and Cultivar Transferability in Machine-Learning Models of Ultrasonication-Induced Functional Properties of Rice Flour
Source: Foods. 2026 Jun 24;15(13):2268. doi: 10.3390/foods15132268 (PMC13361452; doi:10.3390/foods15132268)
Supplement: Supplementary file 1 [file foods-15-02268-s001.zip › Table_S6_Sensitivity_LOCO_no_Weolbaek.pdf]

Table S6. Leave-one-cultivar-out validation performance after excluding Weolbaek (XGBoost).

Held-out cultivar	Response	Model A R^2	Model B R^2	ΔR^2
Saechungmu	WSI	0.578	0.635	0.057
	η_{50}	0.756	0.761	0.006
	Setback	0.717	0.723	0.006
Samgwang	WSI	-0.401	-0.618	-0.217
	η_{50}	0.867	0.749	-0.117
	Setback	0.808	0.733	-0.075
Chamdream	WSI	0.220	0.460	0.241
	η_{50}	0.248	0.534	0.286
	Setback	0.006	0.666	0.659
Seolgang	WSI	-0.701	0.117	0.818
	η_{50}	0.337	0.504	0.167
	Setback	0.099	0.327	0.228
Akibare	WSI	0.582	-0.176	-0.758
	η_{50}	0.924	0.672	-0.252
	Setback	0.901	0.145	-0.756

Note. Model definitions are given in Table 1. ΔR^2 was calculated as R^2(Model B) − R^2(Model A); positive values indicate improvement under Model B. These results are cultivar-level holdout performance from the five-cultivar sensitivity LOCO-CV; for the corresponding six-cultivar LOCO-CV, see Table 3.
