# Supplementary material for: Composition Descriptors and Cultivar Transferability in Machine-Learning Models of Ultrasonication-Induced Functional Properties of Rice Flour
Source: Foods. 2026 Jun 24;15(13):2268. doi: 10.3390/foods15132268 (PMC13361452; doi:10.3390/foods15132268)
Supplement: Supplementary file 1 [file foods-15-02268-s001.zip › Table_S7_cross_fitted_SHAP.pdf]

Table S7. Cross-fitted SHAP feature importance for the within-domain XGBoost Model B.

Response	Variable	Mean |SHAP|	Relative importance (%)
WSI	Time	0.0661	29.6
	Amylose	0.0502	22.4
	Fiber	0.0445	19.9
	Amplitude	0.0315	14.1
	Concentration	0.0222	9.9
	Protein	0.0092	4.1
η_{50}	Time	0.2469	32.9
	Amplitude	0.1846	24.6
	Fiber	0.1059	14.1
	Concentration	0.0852	11.3
	Amylose	0.0645	8.6
	Protein	0.0639	8.5
Setback	Time	56.63	32.4
	Amplitude	37.73	21.6
	Fiber	25.27	14.4
	Protein	21.01	12.0
	Concentration	17.24	9.8
	Amylose	17.17	9.8

Note. SHAP values were computed out-of-fold using cross-fitting for the within-domain XGBoost Model B. Mean |SHAP| is the mean absolute SHAP value in the units of each response (WSI dimensionless, η_{50} Pa·s, Setback cP); relative importance is the normalized mean absolute SHAP value, summing to 100% within each response.
